# Multi-Wavelength Raman Differentiation of Malignant Skin Neoplasms

**DOI:** 10.3390/ijms25137422

**Published:** 2024-07-06

**Authors:** Elena Rimskaya, Alexey Gorevoy, Svetlana Shelygina, Elena Perevedentseva, Alina Timurzieva, Irina Saraeva, Nikolay Melnik, Sergey Kudryashov, Aleksandr Kuchmizhak

**Affiliations:** 1 Lebedev Physical Institute, 119991 Moscow, Russia; rimskaya@lebedev.ru (E.R.); a.gorevoy@lebedev.ru (A.G.); shelyginasn@lebedev.ru (S.S.); perevedencevaev@lebedev.ru (E.P.); a.timurzieva@lebedev.ru (A.T.); saraevain@lebedev.ru (I.S.); melniknn@lebedev.ru (N.M.); kudryashovsi@lebedev.ru (S.K.); 2 Semashko National Research Institute of Public Health, 105064 Moscow, Russia; 3 Institute of Automation and Control Processes, Far Eastern Branch, Russian Academy of Science, 690041 Vladivostok, Russia; 4 Far Eastern Federal University, 690922 Vladivostok, Russia

**Keywords:** basal cell carcinoma, squamous cell carcinoma, Raman microspectroscopy, multispectral analysis

## Abstract

Raman microspectroscopy has become an effective method for analyzing the molecular appearance of biomarkers in skin tissue. For the first time, we acquired in vitro Raman spectra of healthy and malignant skin tissues, including basal cell carcinoma (BCC) and squamous cell carcinoma (SCC), at 532 and 785 nm laser excitation wavelengths in the wavenumber ranges of 900–1800 cm^−1^ and 2800–3100 cm^−1^ and analyzed them to find spectral features for differentiation between the three classes of the samples. The intensity ratios of the bands at 1268, 1336, and 1445 cm^−1^ appeared to be the most reliable criteria for the three-class differentiation at 532 nm excitation, whereas the bands from the higher wavenumber region (2850, 2880, and 2930 cm^−1^) were a robust measure of the increased protein/lipid ratio in the tumors at both excitation wavelengths. Selecting ratios of the three bands from the merged (532 + 785) dataset made it possible to increase the accuracy to 87% for the three classes and reach the specificities for BCC + SCC equal to 87% and 81% for the sensitivities of 95% and 99%, respectively. Development of multi-wavelength excitation Raman spectroscopic techniques provides a versatile non-invasive tool for research of the processes in malignant skin tumors, as well as other forms of cancer.

## 1. Introduction

Malignant skin tumors are a serious health problem in all countries [1,2]. A reduction in the mortality of skin tumors has been achieved primarily through early detection and treatment. Currently, a biopsy is used to definitively diagnose and determine appropriate treatment; however, this requires tissue removal, which may itself alter the progression of the disease. The development of optical methods for noninvasive diagnosis can dramatically improve the detection of skin tumors and help with therapy [3,4]. Despite the huge amount of research, there is still no complete picture of the mechanism of the occurrence and development of tumors, and the role of various biologically active molecules in the pathogenesis of tumor growth has not been sufficiently studied [5,6,7,8].

In recent years, Raman microspectroscopy has become one of the leading optical tools for non-invasive analysis of the molecular composition of skin tissues and detection of biomarkers for early diagnosis of cancer [9]. Pathologies generally do not cause the appearance or disappearance of specific bands in Raman spectra of tissues, but rather, they cause changes in band positions and peak intensities. Different cellular origins of skin cancers lead to distinct Raman spectral features, providing a reliable basis for their differentiation. Thus, it is easier to recognize malignant melanoma derived from melanocytes than to distinguish between basal cell carcinoma (BCC) and squamous cell carcinoma (SCC), both of which stem from malignant keratinocytes. Nevertheless, several studies have demonstrated that Raman spectroscopy can be used for discrimination of normal skin tissues, BCC, and SCC with high sensitivity (>60%) and specificity (>80%) both in vitro and in vivo [6,8,10,11,12,13,14,15]. In a majority of the studies, a high-dimensional space of Raman spectra was transformed into a lower-dimensional space of spectral features using various algorithms, such as standard principal component analysis (PCA) [16,17], multivariate curve resolution (MCR) analysis [4,10], convolutional neural networks (CNN) [7,13,18,19] and others, which make it possible to achieve remarkable classification rates for benign and malignant skin tumors. However, these methods do not always provide meaningful results that can elucidate the nature of biochemical processes responsible for spectral differences in the skin tissues and explain the choice of spectral features. Analogously, a spectral decomposition using a small number of predefined ingredients (collagen, keratin, triolein, etc.) [20,21], as well as the attribution of the MCR-produced components to such ingredients [22], may suffer from the unsuitable choice of the ingredient set and significant correlation between their spectra. In some cases, similar classification performance was achieved using several biochemically representative Raman bands [3], which can be preferable to acquiring and processing full spectra for faster mapping and the detection of tumor boundaries using snapshot multi-spectral imaging or lower spectral resolution. Despite significant progress in this field, the development of new methods for Raman-based diagnostics of non-melanoma malignant skin tumors remains the focus of recent studies [9], including multi-wavelength [3], spatially offset [23], stimulated and coherent anti-Stokes [24] Raman spectroscopy, as well as their combination with autofluorescence [6] and two-photon excited fluorescence [25].

The Raman spectroscopy of malignant skin tissues mostly used near-infrared (NIR) laser excitation (750–850 nm) because of a lower background caused by tissue autoflourescence [26]. However, laser sources in the visible range (488, 514.5, 532 nm, etc.) provide higher Raman intensity and allow for a significant reduction in acquisition time, so they have been used successfully along with NIR sources for colon [16], breast [27], brain [28] and other tissues to diagnose and study tumor development but have been used relatively rarely for skin tumors [3,27,29]. When the excitation wavelength is near the frequency of the electronic transition of tissue chromophores (or overlaps with their absorption band), the intensity of inherently weak Raman bands significantly enhances via coupling of electronic and vibrational transitions, which is known as the resonance Raman enhancement. Among the chromophores of skin tissues, this effect at 532 nm excitation takes place for carotenoids and heme proteins, so it was used to measure carotenoid content and study the porphyrin vibrations of proteins and the oxidation and spine states of the heme iron species [30,31,32,33]. Since cancer is characterized by aberrant heme metabolism [34,35] and may negatively correlate with the carotenoid content [29,36], the combination of visible- and NIR-excited Raman spectroscopy is promising for the examination of molecular biomarkers, the use of which can form the basis of new screening methods to identify signs of malignant growth before the formation of morphological manifestations and provide fast differentiation between the types of tumors.

In this study, we acquire and analyze Raman spectra of normal skin, BCC, and SCC samples using 532 and 785 nm laser excitation in the commonly used fingerprint wavenumber range (900–1800 cm^−1^) of the most optically active components of skin tissues as well as in the less-frequently and separately used higher wavenumber range (2800–3100 cm^−1^), which also contains important spectral features. According to our knowledge, it is the first paper that combines Raman biomarkers for 532 and 785 nm excitation wavelengths in the two wavenumber ranges to show the biochemical difference between normal skin and skin tumors and the advantages of each wavelength and their combined use, allowing for the choice of the best spectral range for differential diagnosis. The proposed approach allows one to find out the similarities in the spectra and to discriminate the spectra exhibiting possible anomalies, thus providing a powerful tool for the analysis of spectra with small differences.

## 2. Results and Discussion

### 2.1. Main Raman Bands

**Figure 1:**
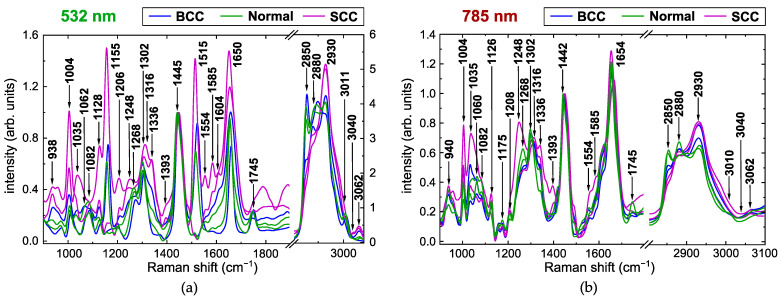
Examples of processed Raman spectra for basal cell carcinoma (BCC, blue curves), normal skin (Normal, green curves), and squamous cell carcinoma (SCC, purple curves) obtained at (**a**) 532 nm and (**b**) 785 nm excitation wavelengths. For each class, averaged spectra for two arbitrarily chosen samples are shown.

**Figure 2:**
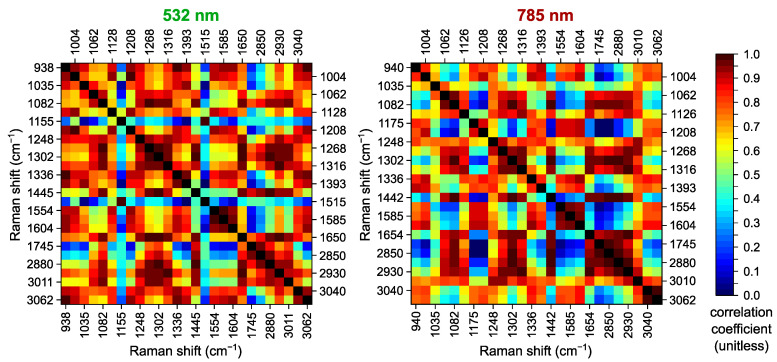
Correlation coefficients for peak intensities of main Raman bands calculated for spectra acquired at 532 nm (**left panel**) and 785 nm (**right panel**) excitation wavelengths.

The processed Raman spectra in the wavenumber ranges of 900–1800 cm^−1^ and 2800–3100 cm^−1^ for all skin tissue samples included in this study (see Section 3 for details) have similar major Raman bands; however, they differ in peak positions and intensities. The representation of each spectrum by an array of its peak intensities *I* in these bands can greatly reduce the amount of excessive data, but the bands should be properly chosen to avoid losing information important for further classification. Therefore, we start our analysis from the detection of prominent peaks in the spectra and search for their possible correspondence to various basic constituents of the skin tissues. Figure 1 demonstrates examples of normalized mean Raman spectra for the samples of healthy skin, BCC and SCC (two samples for each class) at 532 and 785 nm laser excitation with the indicated main Raman bands considered in this work. The possible assignments of these bands to specific vibrations in the molecules of tissue components known from the literature are listed in Table 1. Since many bands with close central wavenumbers can overlap and the assignments of these bands in different works may be controversial, we additionally calculated correlation matrices (see Figure 2) for the arrays *I* of peak intensities to find the potential association of several bands with the same constituent for our datasets.

One of the main differences between the Raman spectra obtained at different excitation wavelengths is the presence of the characteristic bands of intrinsic tissue chromophores, such as carotenoids with three main bands at 1005 cm^−1^ (${\mathrm{CH}}_{3}$ rocking), 1155 cm^−1^ (C–C stretching), and 1515 cm^−1^ (C=C stretching) [43]. These bands may be very intense in the spectra under 532 nm laser excitation (see Figure 1a) because of the broad absorption range of these pigments, with a maximum near 480 nm and the Raman resonance effect [16]. In contrast, the specific bands around 1155 and 1515 cm^−1^ are not visible in the spectra of the same samples at a 785 nm excitation wavelength (Figure 1b). Carotenoids are available in all human tissues, but their content may depend on the individual traits of a person and may be affected by their lifestyle (smoking, fatigue, illness, etc.) [3,43]. As can be seen from the examples given in Figure 1a, the intensity of the corresponding bands, $I(1155\;{\mathrm{cm}}^{-1})$ and $I(1515\;{\mathrm{cm}}^{-1})$, can be very high or negligible for all three classes of samples, indicating low reliability of differentiation between the normal skin and the tumors. Figure 2 (left panel) shows that $I(1155\;{\mathrm{cm}}^{-1})$ and $I(1515\;{\mathrm{cm}}^{-1})$ are strongly correlated (correlation coefficient is 0.99), but the intensity of the third band at 1005 cm^−1^ is mainly determined by other tissue components, which explains its presence in the spectra acquired with 785 nm laser excitation. Additionally, the expressive strengthening of the Raman bands of chromophores at 532 nm may mask characteristic bands of other elements, e.g., the Raman band at 1175 cm^−1^ (mainly tyrosine), which is poorly distinguishable at 532 nm due to the wide band of carotenoids, but can be detected at 785 nm (Figure 1).

Most of the remaining Raman bands can be divided into two big groups according to their possible assignments (Table 1) and the calculated correlation coefficients (Figure 2). The first group is mainly associated with proteins, including characteristic bands of aromatic and aliphatic amino acids and nucleic acids, which are typical for collagen, elastin, keratin, and also the cytoplasm and cell nucleus [20,39]. In the 900–1800 cm^−1^ range, this group comprises the specific bands of phenylalanine (Phe) at 1004, 1035, 1208, 1585, and 1604 cm^−1^ [16,39,40,57,59], demonstrating high correlation coefficients (up to 0.98 for $I(1004\;{\mathrm{cm}}^{-1})$ and $I(1208\;{\mathrm{cm}}^{-1})$ at 532 nm excitation). The rather strong band of Phe at 1004 cm^−1^ overlaps with the band of tryptophan (Trp) at 1009–1012 cm^−1^ [59,60], so $I(1004\;{\mathrm{cm}}^{-1})$ is also correlated with the intensities of other Trp bands, namely $I(1316\;{\mathrm{cm}}^{-1})$, $I(1336\;{\mathrm{cm}}^{-1})$, and $I(1554\;{\mathrm{cm}}^{-1})$ [39,40,57,59]. Some of the mentioned bands are additionally associated with tyrosine (Tyr) [40,57,59], which can explain the significant correlation of $I(1175\;{\mathrm{cm}}^{-1})$ and $I(1208\;{\mathrm{cm}}^{-1})$ at the 785 nm excitation wavelength and increase the coefficient for $I(1604\;{\mathrm{cm}}^{-1})$. In addition to the aromatic amino acids (Phe, Trp, Tyr), the Raman bands at 938, 1128, 1248, 1336, 1393, 1554, and 1604 cm^−1^ are influenced by the content of aliphatic amino acids (proline (Pro), valine, etc.) [16,20] and nucleic acids (adenine, guanine, cytosine) [40,47,61]. The intensities $I(1554\;{\mathrm{cm}}^{-1})$, $I(1585\;{\mathrm{cm}}^{-1})$, and $I(1604\;{\mathrm{cm}}^{-1})$ of the neighboring bands present in many protein components are characterized by remarkable correlation coefficients (0.94–0.98) at both excitation wavelengths. Moreover, these bands, as well as the ones at 1128, 1316, and 1393 cm^−1^, can be related to myoglobin (Mb) [16], hemoglobin (Hb), cytochromes (Cyt) and other heme proteins, for which resonance Raman enhancement due to the electronic absorption of porphyrins is strong at 532 nm [39]. Finally, the prominent Raman bands at 1248, 1268, and 1650 cm^−1^ in the spectra of skin tissues are attributed to the amide III and amide I bands of proteins in the α-helix conformation [39,45]. Thus, in the vibrational spectra of many proteins, e.g., albumin, the vibrations of the amide group of polypeptides can be distinguished, the characteristic frequencies of which lie in the spectral ranges near 1248 and 1650 cm^−1^, and the vibration modes are determined by changes in the length of the C=O peptide bond and the length of the C–N [39,40,57].

The Raman bands of the other group are mostly associated with lipids that can be found in skin tissues in various forms (triglycerides, wax esters, squalene, ceramides, fatty acids, cholesterol, etc. [20,41]). Generally, the Raman spectra for phospholipids, triacylglycerols and membrane lipids in the fingerprint (900–1900 cm^−1^) spectral region are quite similar and contain Raman bands derived from the C=O ester stretching vibrations (1745 cm^−1^), C=C stretching vibrations of unsaturated fatty acids (1650 cm^−1^), CH_2_ scissoring mode (1445 cm^−1^), CH_2_ twisting mode (1302 cm^−1^) and =C–H in-plane deformation vibrations (1268 cm^−1^) [16,41]. Phospholipids with a clearly separated band at 1082 cm^−1^ (gauche C–C stretching) are present in the spectra of other lipids. As a result, correlation coefficients for *I*(1082 cm^−1^), *I*(1302 cm^−1^), *I*(1445 cm^−1^), and *I*(1650 cm^−1^) are considerably high (0.96–0.99), indicating the main lipid-associated bands (see Figure 2). In addition, the bands at 1062 and 1082 cm^−1^ are closely related due to the broad peak in the spectra of many fatty acids, and the bands at 1268 and 1302 cm^−1^ are noted together in the spectra of fatty acids and triacylglycerols (e.g., triolein) [20,41]. The band at 1128 cm^−1^ is also attributed to C–C and C–N stretching in the molecules of triacylglycerols and phospholipids [40,41], which is evidenced by increased coefficients for *I*(1128 cm^−1^) and other mentioned bands corresponding to lipids.

The higher wavenumber range (2800–3100 cm^−1^) covers a very intense group of bands caused by ${\mathrm{CH}}_{2}$ (2832–2888 cm^−1^) and ${\mathrm{CH}}_{3}$ (2909–2967 cm^−1^) symmetric and asymmetric stretching in lipids at 2850, 2880, and 2930 cm^−1^ [41]. As can be seen in Figure 2, the correlation between the intensities of these bands and the main lipid-associated bands is significant, including a high coefficient for $I(2850\;{\mathrm{cm}}^{-1})$ and ester-related $I(1745\;{\mathrm{cm}}^{-1})$. The band at 3010 cm^−1^ falls in the 3000–3015 cm^−1^ range assigned to =C–H stretching vibrations, which indicates the lipid unsaturation together with 1268 (=C–H deformation) and 1650 cm^−1^ (C=C stretching) bands, accompanied by gauche C–C stretching at 1082 cm^−1^ [41,56]. At the same time, the features of lipid components in the 2800–3100 cm^−1^ spectral range considerably overlap with similar CH_2_ and CH_3_ stretching bands of protein components. Generally, the bands close to 2850 cm^−1^ are more affected by the content of lipids, whereas the bands close to 2930 cm^−1^ are more influenced by proteins [56,62], as evidenced by higher correlation coefficients for *I*(2930 cm^−1^) and protein-associated bands, e.g., *I*(938 cm^−1^), in Figure 2 (left panel). Furthermore, the bands at 2930 and 3062 cm^−1^ are very intense in the Raman spectra of all three aromatic amino acids (Phe, Tyr, Trp), while *I*(2850 cm^−1^) is quite weak for Phe and Trp and indistinguishable for Tyr [57]. A high coefficient (0.93 and 0.99 for 532 and 785 nm, respectively) for *I*(3040 cm^−1^) and *I*(3062 cm^−1^) may be explained by their presence in the spectra of Phe [57] and their proximity, so both bands can be included in the protein-assigned group.

Although many of the specified Raman bands can be classified into either protein- or lipid-associated groups, some of them are almost equally affected by both components because of similar molecular vibrations and broad overlapping peaks. Thus, the bands at 1248, 1268, 1302, and 1316 cm^−1^ demonstrate an example of this complex relationship, which can be seen in the spectra at 532 nm excitation (Figure 1a) as follows. The spectra of normal skin samples (green curves) are characteristic of lipids with clearly visible peaks at 1302 and 1268 cm^−1^. Although the spectra of SCC samples (purple curves) are a distinct manifestation of proteins with peaks at 1248, 1316 and 1336 ${\mathrm{cm}}^{-1}$, the intensities $I(1268\;{\mathrm{cm}}^{-1})$ and $I(1302\;{\mathrm{cm}}^{-1})$ are still quite high due to overlapping, which leads to their significant correlation (Figure 2). A similar picture can be observed for the bands at 2850, 2880, and 2930 cm^−1^ for both excitation wavelengths, which allows us to label these bands as mostly lipids (2850), mixed (2880), and mostly proteins (2930). According to the correlation matrices shown in Figure 2, the band at 1650 cm^−1^ is closer to the lipid-related bands, but the contribution of proteins is also noticeable because the corresponding coefficients are considerably higher than for the neighboring ones $I(1745\;{\mathrm{cm}}^{-1})$ (only lipids). Analogously, in the analyzed dataset, the impact of lipids is predominant for the band at 1445 ${\mathrm{cm}}^{-1}$, associated with both lipids and proteins (${\mathrm{CH}}_{2}$ and ${\mathrm{CH}}_{3}$ bending and scissoring [16,41], C–H deformation [39]), especially for the 785 nm laser excitation.

**Figure 3:**
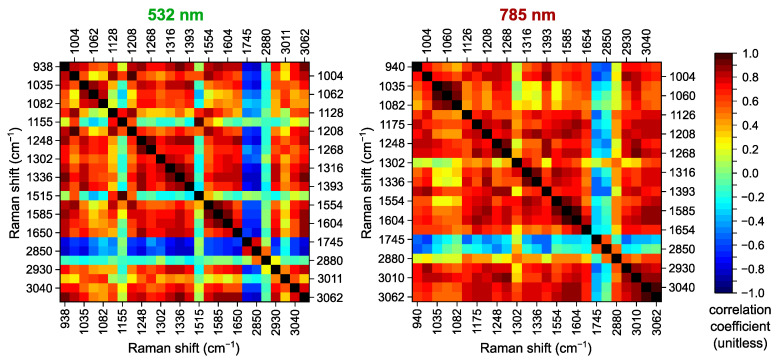
Correlation coefficients for normalized peak intensities of main Raman bands calculated for spectra acquired at 532 nm (**left panel**) and 785 nm (**right panel**) excitation after division by peak intensities of 1445 cm^−1^ and 1442 cm^−1^ bands, respectively.

The band at 1445 cm^−1^ is relatively conformation-insensitive, being a characteristic feature of typical Raman spectra for both tumors and healthy skin, and therefore, it has been proposed as an intensity standard and used for normalization to obtain ratios of intensities for the remaining Raman bands [3,16]. In our case, the division by $I(1445\;{\mathrm{cm}}^{-1})$ and $I(1442\;{\mathrm{cm}}^{-1})$ for the datasets acquired at 532 and 785 nm excitation, respectively, corresponds to the division by the intensity of the band mostly affected by lipids and, therefore, allows us to reveal relative changes in the content of different lipid components and obtain the proteins/lipids ratios. The correlation matrices for the intensities of the main Raman bands after division are presented in Figure 3. One can see that $R(1745\;{\mathrm{cm}}^{-1})$ ($R\left(1745\;{\mathrm{cm}}^{-1}\right)=I\left(1745\;{\mathrm{cm}}^{-1}\right)/I\left(1445\;{\mathrm{cm}}^{-1}\right)$, see Section 3 for notation) and $R(2850\;{\mathrm{cm}}^{-1})$, assigned to lipids only, now have negative correlation coefficients (reaching −0.83 and −0.58 at 532 and 785 nm, respectively) with almost all other bands excluding carotenoid-related ones. Thus, the changes in the intensities of the Raman bands at 2850 and 2930 cm^−1^ have opposite directions, which can be seen in Figure 1 and the spectra presented in other works [11,51]. Consequently, the correlation of $R(1248\;{\mathrm{cm}}^{-1})$ and $R(1268\;{\mathrm{cm}}^{-1})$, as well as $R(1316\;{\mathrm{cm}}^{-1})$ and $R(1336\;{\mathrm{cm}}^{-1})$, caused by vibrations of protein molecules becomes clearly visible since the impact of lipid-related features at 1268 and 1316 cm^−1^ diminishes. Interestingly, the mixed band at 2880 cm^−1^ has close to zero or slightly positive coefficients with most of the other bands except for the neighboring band at 2850 cm^−1^. As a result, the impact of normalization on mixed and overlapping bands and derived spectral features may be meaningful for the subsequent differentiation between the normal skin and the tumors, so it will be analyzed in detail in Section 2.4.

### 2.2. Comparison of Spectral Feature Extraction Methods

Following the proposed scheme for selecting the best spectral features to differentiate between three classes of samples (BCC, normal skin, and SCC), we applied three methods of feature extraction (PCA spectra, PCA bands, and Selected bands, see Section 3 for details) to the normalized Raman spectra and the arrays *R* of intensity ratios. The calculated true positive rate (TPR) values for each class, as well as for all classes together (All), are listed in Table 2 for different numbers *N* of selected principal components or intensity ratios. As can be seen for the PCA of normalized Raman spectra (PCA spectra), using just two components (N=2) provided a quite high overall TPR of ≈80% with >90% for SCC; increasing the number of components to five allowed us to obtain a ≈95% rate of correct recognition for each of three classes. We should note that the selected principal components with the best TPR were not the most significant ones when sorted according to a decrease in eigenvalues (e.g., the second and the seventh ones), so the first ones described the variations in spectra due to individual characteristics of patients that are not significant for the classification of tumors. Generally, the PCA of the spectra acquired at 532 nm laser excitation produced spectral features with higher overall TPR than the 785 nm dataset, which was mainly due to the reduced ability of BCC recognition for the 785 nm one, especially pronounced for N<4. Consequently, the best features for the merged dataset (532 + 785 in Table 2) were mostly chosen from the components of the 532 nm spectra, but in some cases (e.g., for N=4), adding the features from the 785 nm dataset could slightly improve the overall TPR.

A transition from the PCA of the spectra to the PCA of the arrays *R* of intensity ratios for the main Raman bands (PCA bands) did not lead to a meaningful decrease in the overall TPR; hence, all relevant bands were present in the datasets containing intensity ratios. At the same time, the PCA bands method gave a lower TPR for BCC but a higher TPR for normal skin than PCA spectra. An impressive TPR for a small number of selected principal components, reaching 86–88% for just three of them, along with high correlation coefficients for most of the considered bands (see Figure 3) suggests that a similar result can be achieved using a small number of carefully selected representative Raman bands. Indeed, choosing the same number of ratios *R* as the number of principal components allowed us to obtain comparable or even better overall TPR values in all cases, including 86.6% for the merged dataset with N=3. For N>2, selecting the ratios from the merged dataset (532 + 785) made it possible to improve the classification performance compared to the 532 nm one; therefore, in some Raman bands, the spectral features of the characteristic constituents of the malignant tumors were more distinct at 785 nm laser excitation.

**Figure 4:**
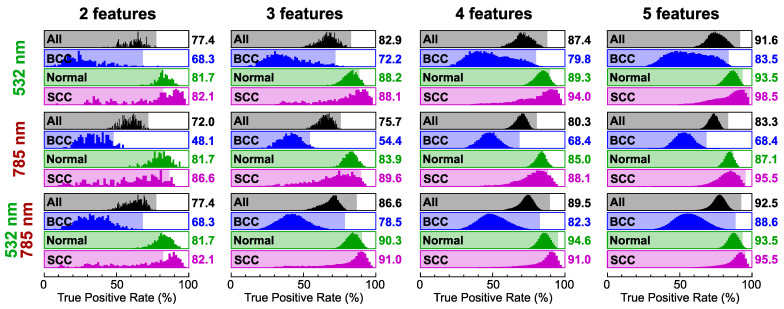
Histograms of true positive rate (TPR) values for BCC, normal skin (Normal), SCC, and all three classes (All) calculated for different numbers of selected spectral features (as indicated in the top part of each column) using data acquired at excitation wavelengths of 532 nm (**top row**) and 785 nm (**middle row**) as well as at both wavelengths (**bottom row**). The results for the set of features with the highest TPR for all classes are indicated by background color bars and values on the right side of each histogram.

In order to estimate the number of possible sets of *N* Raman bands providing high classification rates, we tried all possible combinations and analyzed the histograms of TPR for each class and all datasets, as presented in Figure 4. According to the results for the overall TPR, the mean value was 10–20% less than the maximum, although up to 10 best sets could have TPR values within 2%, especially for N=5. Since the best sets were determined by the overall TPR value, they did not necessarily provide the maximum values of TPR for each class. Basically, the correct classification of BCC was the most problematic, so the selected sets also had some of the highest rates for BCC. In contrast, a lot of sets can be chosen to obtain TPR values exceeding 90% for the two remaining classes, especially SCC, so the feature selection algorithm can be tuned in accordance with the required diagnostic performance in terms of sensibility and specificity for the normal skin and the tumors. These attributes are considered in the detailed analysis of the selected Raman bands in the next section including distributions of spectral features and receiver operating characteristic (ROC) curves.

### 2.3. Detailed Analysis of Selected Raman Bands

**Figure 5:**
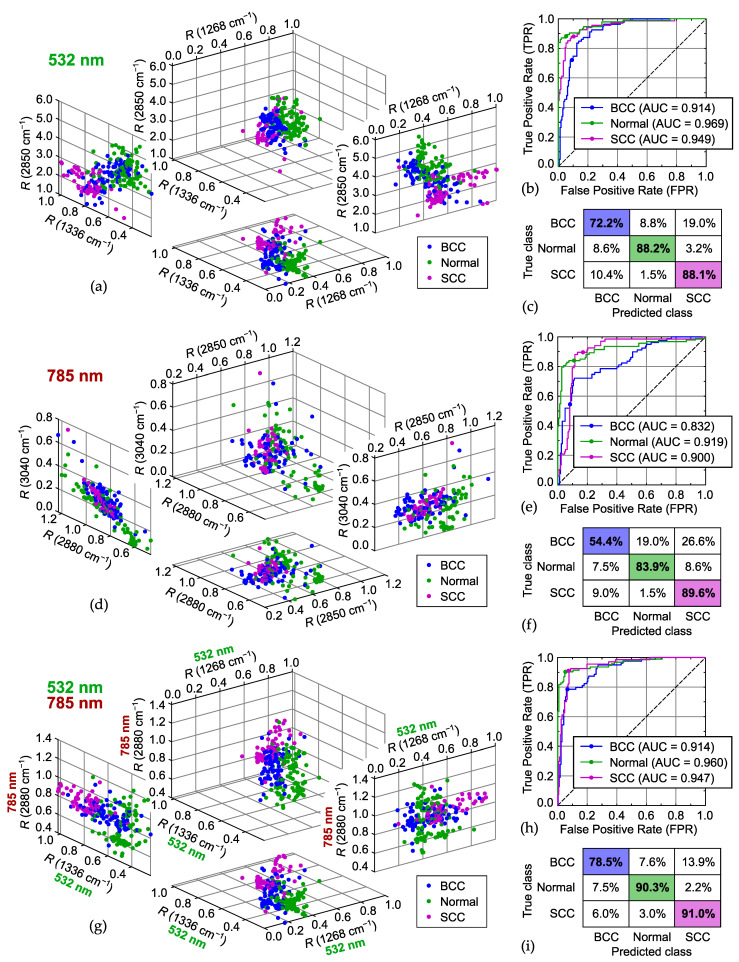
Results of classification (BCC, Normal, SCC) obtained for datasets acquired at (**a**–**c**) 532 nm, (**d**–**f**) 785 nm, and (**g**–**i**) both excitation wavelengths: (**a**,**d**,**g**) distribution of normalized intensity values for three selected Raman bands, (**b**,**e**,**h**) receiver operating characteristic (ROC) curves (filled circle on each plot indicates selected working point), and (**c**,**f**,**i**) confusion matrices.

An analysis of the most representative Raman bands that provide the highest classification scores is useful for understanding the biochemical changes during tumor development, where protein and lipid contents can play an important role as biomarkers. The results of the study considering 53 bands at two excitation wavelengths demonstrated that there were specific differences in their relative intensities for the malignant tumors (BCC, SCC) compared with the normal skin. The distributions of the three best spectral features (intensity ratios *R*) calculated for the datasets acquired at 532 nm, 785 nm and both excitation wavelengths (combined) are shown in Figure 5, as well as ROC curves and confusion matrices. The case of three features (N=3) was chosen as the most suitable for graphical illustration, and the others are described in the text along with the best sets listed in Table 3.

The results of differentiation between the normal skin and the tumors using the 532 nm dataset demonstrated that the bands at 1248, 1268, and 1336 cm^−1^ are among the most effective with an overall TPR of 77.4% for two of these bands and 82.4% for three of them (see Table 3). The intensity ratios *R* for all three bands are increased for the SCC, so each of them could be used to recognize it. As can be seen in Figure 5a, the normal skin samples are characterized by higher proportions $R\left(1268\;{\mathrm{cm}}^{-1}\right)/R\left(1336\;{\mathrm{cm}}^{-1}\right)$ or $R\left(1248\;{\mathrm{cm}}^{-1}\right)/R\left(1336\;{\mathrm{cm}}^{-1}\right)$, which made it possible to separate them from the samples of the both tumors. A similar criterion was used to distinguish normal skin from the BCC in [13], where the reduced Raman intensity at 1268 cm^−1^ was explained by alteration of the α-helix structure of proteins in the BCC. Moreover, the decrease in intensity at 1248 and 1268 cm^−1^ was associated with a significantly lower content of collagen in the BCC and SCC caused by the role metalloproteinases play in degrading collagen and prohibiting procollagen biosynthesis [20,21,63]. At the same time, an excessive $R(1268\;{\mathrm{cm}}^{-1})$ together with $R(1302\;{\mathrm{cm}}^{-1})$ can be related to a higher content of lipids (triglycerides) in the normal skin, but their impact is conceivably diminished because of division on $I(1445\;{\mathrm{cm}}^{-1})$, mainly influenced by lipids. Similarly, the band at 1062 cm^−1^ present in the spectra of lipids can be used instead of 1248 or 1268 cm^−1^ without loss in the overall TPR since $R\left(1062\;{\mathrm{cm}}^{-1}\right)/R\left(1336\;{\mathrm{cm}}^{-1}\right)$ is also greater for the normal skin tissue. The rise of $R(1336\;{\mathrm{cm}}^{-1})$ can indicate the increased content of nucleic acids in the BCC due to higher cell density [20,63] or the elevated percentage of tryptophan content relative to the total Raman-active components in the BCC and SCC samples. For the SCC, a possible explanation of the growth of $R(1336\;{\mathrm{cm}}^{-1})$ is massive keratinization disorders during tumor progression and, consequently, a substantially larger amount of keratin [20,21,64]. In accordance with the significant correlation coefficient for $R(1248\;{\mathrm{cm}}^{-1})$ and $R(1268\;{\mathrm{cm}}^{-1})$ (Figure 3), they are interchangeable as discriminating criteria for N=2 and some sets with N>2 (Table 3). However, $R(1248\;{\mathrm{cm}}^{-1})$ and $R(1268\;{\mathrm{cm}}^{-1})$ can be used together to distinguish the normal tissues from the cancerous ones owing to slightly increased $R\left(1268\;{\mathrm{cm}}^{-1}\right)/R\left(1248\;{\mathrm{cm}}^{-1}\right)$ similar to $R\left(1268\;{\mathrm{cm}}^{-1}\right)/R\left(1336\;{\mathrm{cm}}^{-1}\right)$. The Raman bands at 1554, 1585, and 1604 cm^−1^, detected in many protein-rich components, including keratin and heme proteins [10,16,20], and marked by significant mutual correlation coefficients, demonstrate similar trends with excessive values of *R* for the SCC samples. Therefore, they can be added to the best bands for N=3 to form the sets with N>3 and improve overall TPR values, which is also determined by the fact that the fractions $R\left(1268\;{\mathrm{cm}}^{-1}\right)/R\left(1585\;{\mathrm{cm}}^{-1}\right)$ and $R\left(1585\;{\mathrm{cm}}^{-1}\right)/R\left(1336\;{\mathrm{cm}}^{-1}\right)$ are greater for the normal skin. Additionally, the band at 1004 cm^−1^ (Phe and overlapped band of Trp) is also strong in the spectrum of keratin [20,21,64] and indicates an increased content of Phe in the SCC [14], so $R(1004\;{\mathrm{cm}}^{-1})$ is unsurprisingly higher in the spectra of the SCC samples and lower for the normal tissues and, for this reason, appears in the best sets as well.

The higher wavenumber range also contains reliable indicators of the disturbed protein–lipid ratio in the tissues of the malignant tumors, representing symmetric and antisymmetric stretching vibrations of methyl and methylene groups of lipids and proteins [11,41,51]. The intensities of the bands at 2850 cm^−1^ (mostly lipids) and 2930 cm^−1^ (mostly proteins), exhibiting a negative correlation coefficient, specify that the relative amount of proteins (presumably keratin [65]) grows significantly in the SCC samples. Thus, Figure 5a demonstrates the increased values of $R(2850\;{\mathrm{cm}}^{-1})$ for the normal tissue and the decreased ones for the SCC, which makes it possible to separate them with high probability. In combination with the bands at 1248, 1268, and 1336 cm^−1^, the intensity ratios $R(2850\;{\mathrm{cm}}^{-1})$ and $R(2930\;{\mathrm{cm}}^{-1})$ compose sets with top overall TPR values (see sets with N>2 in Table 3). The best set of three bands has an overall TPR of 82.9% for three classes (BCC, Normal skin, and SCC); its ROC curves and confusion matrix are shown in Figure 5b,c. For two classes (Normal skin, BCC + SCC), the overall TPR (accuracy) is 90%, the ROC area under the curve (AUC) score is 0.97, and the specificities for BCC + SCC are 87% and 81% for the sensitivities of 95% and 99%, respectively. However, using only the bands from the higher wavenumber range does not provide distinct criteria for the recognition of the BCC, so the overall TPR (three classes) drops down to 66.5% for $R(2850\;{\mathrm{cm}}^{-1})$ and $R(2930\;{\mathrm{cm}}^{-1})$ compared to 77.4% for $R(1268\;{\mathrm{cm}}^{-1})$ and $R(1336\;{\mathrm{cm}}^{-1})$. For example, the distribution of $R(2850\;{\mathrm{cm}}^{-1})$ and $R(2930\;{\mathrm{cm}}^{-1})$ is similar to the distribution of $R(2850\;{\mathrm{cm}}^{-1})$ and $R(1336\;{\mathrm{cm}}^{-1})$ shown in Figure 5a, where the points denoting the BCC overlap with the points of the two other classes without a distinct boundary. The band at 2880 cm^−1^, influenced by both lipids and proteins, occupies an intermediate position in the chain $R\left(2850\;{\mathrm{cm}}^{-1}\right)<R\left(2880\;{\mathrm{cm}}^{-1}\right)<R\left(2930\;{\mathrm{cm}}^{-1}\right)$ specific for the SCC (and vice versa for the normal skin); the ratio $R\left(2880\;{\mathrm{cm}}^{-1}\right)/R\left(2850\;{\mathrm{cm}}^{-1}\right)$ with the excluded impact of keratin characterizes lateral packing of lipids [65]. Finally, $R(3040\;{\mathrm{cm}}^{-1})$ and $R(3062\;{\mathrm{cm}}^{-1})$ are interchangeable due to the remarkably high correlation coefficient (see Figure 3) and represent mostly aromatic amino acids in proteins [42,57], so they act as a replacement for $R(2930\;{\mathrm{cm}}^{-1})$ in the list of sets with N>3 in Table 3.

In contrast, the results obtained using the 785 nm dataset show that the bands at 1248, 1268, and 1336 cm^−1^ do not provide clear differentiation of the normal skin and the tumors. Although the ratio $R\left(1268\;{\mathrm{cm}}^{-1}\right)/R\left(1336\;{\mathrm{cm}}^{-1}\right)$ is still generally greater for the normal tissues, many malignant tumor samples can not be distinguished from them because of lower $R(1336\;{\mathrm{cm}}^{-1})$ values for the BCC and, especially, the SCC compared to the 532 nm dataset. Similarly, the bands at 1554, 1585, and 1604 cm^−1^ are not a clear indication of the SCC samples with excessive intensity values, as they were for the 532 nm excitation. The higher wavenumber range demonstrates the same tendencies revealing a greater amount of lipids in the normal tissues (see the distribution of $R(2850\;{\mathrm{cm}}^{-1})$ in Figure 5d). In comparison with the 532 nm dataset, the intensities at 2930, 3040, and 3062 cm^−1^ have closer values for the spectra of the SCC and BCC samples, but, at the same time, the values for both malignant tumors significantly exceed the values for the normal skin. Thus, such combinations as $R(2880\;{\mathrm{cm}}^{-1})$ and $R(3040\;{\mathrm{cm}}^{-1})$ (or $R(3062\;{\mathrm{cm}}^{-1})$, see Table 3 for N=2) have a low TPR value for the BCC (<50%), which is confused with the SCC, and as a result, a lower overall TPR for the three classes (72%), whereas their classification performance for two classes (Normal skin, BCC + SCC) is considerably better. Indeed, the overall TPR (accuracy) for the two classes reaches 87.5% when using $R(2880\;{\mathrm{cm}}^{-1})$ and $R(3040\;{\mathrm{cm}}^{-1})$ from the 785 nm dataset, which is equivalent to the accuracy of $R(1268\;{\mathrm{cm}}^{-1})$ and $R(1336\;{\mathrm{cm}}^{-1})$ for the 532 nm excitation. The classification scores for the best set with N=3 at 785 nm excitation (Figure 5d–f) for the two classes are as follows: the accuracy is 89%, the ROC AUC is 0.91, and the specificity for BCC + SCC is 81% for the sensitivity of 95%. The band at 940 cm^−1^, appearing in the best sets with N>2, is specific to proteins [37,39] and strong in the spectrum of collagen [20,21], so the $R\left(940\;{\mathrm{cm}}^{-1}\right)/R\left(2930\;{\mathrm{cm}}^{-1}\right)$ ratio is increased in the spectra of the normal tissues and decreased in the SCC-related ones. On the other hand, the band at 1654 cm^−1^, associated with keratin, is intense in the spectra of the SCC. Interestingly, $R(940\;{\mathrm{cm}}^{-1})$ can be replaced by $R(1208\;{\mathrm{cm}}^{-1})$ (Phe, Trp [40,59]), and $R(2930\;{\mathrm{cm}}^{-1})$ alternates with $R(1393\;{\mathrm{cm}}^{-1})$ (leucine [49] and Trp [66] also correlate with the bands at 1004, 1248, and 1268 cm^−1^, as shown in Figure 3, right panel) in the sets with N>3.

Summarizing the results for the two excitation wavelengths, we conclude that the best sets for 532 nm include the bands at 1248 (or 1268) and 1336 cm^−1^, whereas the top sets for 785 nm mainly consist of the bands from the higher wavenumber range and the band at 940 cm^−1^. Consequently, the sets with the highest TPR from the merged (532 + 785) dataset comprise $R(1248\;{\mathrm{cm}}^{-1})$, $R(1268\;{\mathrm{cm}}^{-1})$, and $R(1336\;{\mathrm{cm}}^{-1})$, acquired at 532 nm complemented by intensity ratios from the higher wavenumber range of the spectra measured with the 785 nm excitation. As can be seen in Figure 5g–i, the best set with N=3 (1268 and 1336 cm^−1^ at 532 nm; 2880 cm^−1^ at 785 nm) has a better overall TPR for the three classes (86.6% versus 82.9% for the 532 nm dataset), which is mainly caused by better separation of the BCC and SCC samples. For the two classes, it retains the same characteristics as the best set with N=3 from the 532 nm dataset. Similarly, combining the spectra measured at the two excitation wavelengths allows for slightly higher TPR values (three classes) when using the sets with N>3.

**Figure 6:**
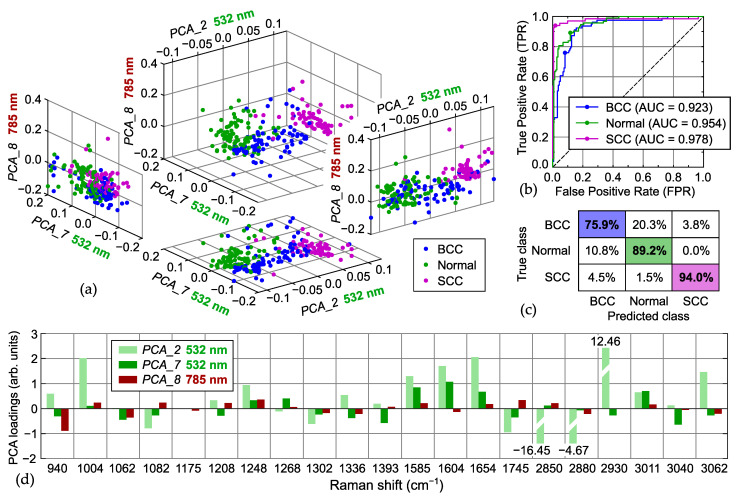
Results of classification (BCC, Normal, SCC) obtained for three principal component analysis (PCA) features (PCA_2, PCA_7, PCA_8) with the highest TPR using the data acquired at both excitation wavelengths (532 nm, 785 nm): (**a**) distribution of PCA coefficients (values), (**b**) ROC curves (filled circle on each plot indicates selected working point), (**c**) confusion matrices, and (**d**) PCA features (loadings).

Similar dependencies are observed for the differentiation performed using the PCA of the intensity ratios for the main Raman bands (PCA bands). Thus, the best set of three features consists of two components (PCA_2 and PCA_7) from the 532 nm dataset and PCA_8 from the 785 nm one; its distributions and performance characteristics are shown in Figure 6. We can deduce that PCA_2 represents a measure of lipid–protein proportion with negative weights for lipid-related bands and positive weights for protein-associated ones (PCA loadings in Figure 6d). This division of bands into two groups is in very good agreement with the division based on the correlation analysis in Section 2.1 (see also Figure 2 and Figure 3) and the results of the PCA spectra (see Figure S1 in Supplementary Materials). According to the distributions in Figure 6a, PCA_2 confirms the protein abundance in the SCC samples and can be used as a criterion to recognize them. Next, PCA_7 allows to differentiate between the normal skin tissues and the BCC, since the latter predominantly has negative values of PCA_7. In addition to lipid–protein content, a detailed analysis of the weights of PCA_7 explains the selection and interchangeability of bands that form the sets with high TPR from the 532 nm dataset (Table 3), e.g., a combination of 1248 or 1268 cm^−1^ (positive weight) and 1336 cm^−1^ (negative weight). Thus, the weights of PCA_7 can be used to further divide the protein-assigned bands into two groups, depending on their intensification in the spectra of the malignant tumors. Finally, PCA_8 from the 785 nm dataset assists in the separation of the samples of tumors providing positive values for the SCC. As a result, the analyzed set of three PCA features (Figure 6b,c) has a higher TPR for the SCC but a lower TPR for the normal skin compared to the best set of three selected bands (Figure 5h,i), which leads to decreased performance for the two classes: 87% accuracy, 0.96 ROC AUC, and 76% specificity for BCC + SCC with 95% sensitivity.

The most significant difference between the data acquired at the two excitation wavelengths is the excessive intensity of the protein-related bands at 1004, 1128, 1316, 1336, 1393, 1554, 1585, 1604, 1650, 2930, and 3062 cm^−1^ in the spectra of the SCC samples at 532 nm, which made it possible to separate them from the other two classes. In addition to the carotenoids, whose content was found to be an unreliable classification criterion in our study, this resonance-enhanced Raman scattering may be associated with the heme proteins (Hb, Mb, Cyt, etc.) [39]. Thus, similar differences between the spectra of the ex vivo human breast cancer tissues at 532 and 785 nm were explained by the influence of Cyt b and c with the bands at 1126, 1300, 1310, 1337, 1398, 1584, and 1632 cm^−1^ [27,28]. According to the conclusions of these works, the mitochondrial content of Cyt is critical in the correct breast ductal functioning, and the quantification of cytochrome redox status can characterize human breast cancer. Oxy-Mb and Deoxy-Mb were among the main chromophores distinguishing the spectra of normal and abnormal colon tissues at 532 and 785 nm with the specific bands at 1129, 1340, 1362, 1396, 1554, 1587, 1605, and 1641 cm^−1^ [16]. Moreover, it was found that human epithelial tumors (breast, lung, ovary, and colon carcinomas) expressed high levels of Mb from the earliest studies of disease developments [67], and Mb expression in normal tissues was significantly lower compared to tumors or recurrent cancers for head and neck SCC [68]. The similarity in the aforementioned main bands of Mb and Cyt, as well as Hb [69], does not allow us to draw specific conclusions from the results of our study, so possible heme-related markers of the SCC are a subject of further investigation. Consequently, we note that the acquisition of Raman spectra of normal and cancerous skin tissues using 532 nm excitation can provide new insights into tumor development and increase the differentiation rate, complementing commonly used 750–850 nm excitation wavelengths.

### 2.4. Impact of Normalization

**Figure 7:**
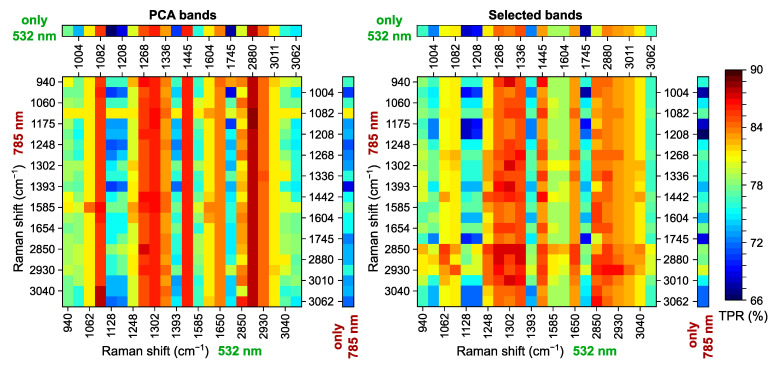
Highest TPR for sets of three selected principle components (**left panel**, PCA bands) and Raman bands (**right panel**, Selected bands) calculated using different bands (as indicated near each row and column) for data normalization at both excitation wavelengths (532 nm, 785 nm, data matrix in the center) and single excitation wavelength (532 nm, top horizontal array; 785 nm, right vertical array).

The results described in Section 2.2 and Section 2.3 were obtained for the peak intensities of the main Raman bands divided by the intensity of the band at 1445 or 1442 cm^−1^. In order to assess the impact of the choice of the band for normalization, we repeated this analysis using the intensity of each band in turn as the denominator to calculate an array of intensity ratios. Similar to the previous section, the case of N=3 is discussed in detail below. The highest overall TPR values are shown in Figure 7 for the best set of three PCA components (PCA bands) and three intensity ratios (Selected bands). A matrix in the center of each panel visualizes the TPR values for the merged (532 + 785) dataset as pseudocolors according to the color bar in the right part of the figure; the bands used for the normalization are labeled near each column (for the data acquired at 532 nm excitation) and each row (at 785 nm excitation). A horizontal array of color pixels at the top and a vertical array in the right part of each panel correspond to the results obtained using only the 532 nm dataset and only the 785 nm dataset, respectively.

The distribution of pseudocolors in the matrix for PCA bands (left panel) indicates that the normalization of the data acquired at 532 nm excitation has a greater effect on the result since the components are mostly selected from this dataset. For 532 nm, the classification rate similar to the default normalization band at 1445 cm^−1^ was achieved for the bands at 1082, 1268, and 1302 cm^−1^, which all have high mutual correlation coefficients (see Figure 2, left panel) and are associated with both lipids and proteins (with a presumably stronger influence of triglycerides caused by their large Raman scattering cross-section [20]). The band at 1650 cm^−1^, also characterized by significant correlation with the aforementioned bands, is less suitable for the normalization because its strength and location fluctuate more for both proteins (amide I) due to alterations in protein secondary structure [39,57] and lipids due to isomerization and variations of saturation level [41] and can also be affected by the content of urocanic acid in the skin tissue [70,71]. A small improvement in the results was achieved by the normalization using the mixed protein–lipid band at 2880 cm^−1^ (≈2% higher than 1445 cm^−1^), the correlation coefficient of which is 0.99 with the band at 1450 cm^−1^. Using the protein-assigned bands at 1336 and 2930 cm^−1^ leads to lower but acceptable TPR values. For 785 nm, the normalization band at 1082 cm^−1^ appeared to be the most stable in terms of classification performance.

The results for Selected bands (right panel of Figure 7) follow similar tendencies as for PCA bands, but the impact of the data acquired at the 785 nm excitation wavelength is more pronounced. The bands at 1268, 1302, and 1336 cm^−1^ from the 532 nm dataset have similar top TPR values, slightly outperforming the default normalization band at 1445 cm^−1^. This was expected because the ratio of intensities for 1268 and 1336 cm^−1^ was among the best criteria of differentiation in Section 2.3. Moreover, the analysis of the bands forming the best sets (see Table 4) reveals that the bands at 1268, 1336, and 1445 cm^−1^ replace each other when one of them is used as the normalization band, accompanied by the Phe + Trp band at 1208 cm^−1^, which is inappropriate for the normalization, and the bands from the higher wavenumber range (2850, 2880, and 2930 cm^−1^). The latter ones are definitely the best choice for the normalization of the 785 nm dataset, allowing an improvement to the TPR by up to 4% compared to 1442 cm^−1^. However, for the merged dataset, the highest TPR achieved using a combination of 1268 and 2880 cm^−1^ for 532 and 785 nm, respectively, does not significantly exceed the result of the default one (1445 and 1442 cm^−1^). Quite interestingly, the band at 2930 cm^−1^ can be used for the normalization of data at both excitation wavelengths with the same overall TPR as 1445 cm^−1^ but lower TPR for normal skin tissues. As a result, in addition to the band at 1445 cm^−1^ proposed by other researchers, we can suggest using the bands at 2880 and 2930 cm^−1^ for the normalization universally for different excitation wavelengths with minor changes in the classification rate, whereas for 532 nm excitation, this list can be complemented by the most informative bands at 1268 and 1336 cm^−1^. The analysis conducted confirms that the most representative bands selected in Section 2.3 and the subsequent conclusions remain valid, and similar classification performance can be achieved using different bands for the data normalization.

## 3. Materials and Methods

Fresh, frozen human malignant and healthy skin tissue samples were acquired from biopsy specimens during routine skin cancer surgical procedures at Sechenov First Moscow State Medical University, Evdokimov Moscow State University of Medicine and Dentistry, and Semashko National Research Institute of Public Health and stored in hermetically sealed cuvettes. In total, 32 BCC, 30 SCC, and 40 normal skin samples measuring 2–7 mm were collected and classified according to the results of histopathological analysis. For spectral measurements, the samples were thawed at room temperature (25 °C) and placed in Petri dishes with saline solution (0.9% NaCl). We do not expect that the sample preparation affected the results of measurements since the previous studies [58,72] confirmed that Raman spectra of frozen tissues did not show any changes from fresh tissue spectra.

The spectra were measured by a Renishaw inVia Basis confocal microscope-spectrometer (inVia InSpect, Renishaw, London, UK) at a 785 nm excitation wavelength (45 mW laser power in the sample plane, 20 s acquisition time) using a 50× objective lens N Plan 50/0.50 (Leica, Wetzlar, Germany) as well as by a Confotec MR520 confocal microscope-spectrometer (SOL instruments, Minsk, Belarus) at 532 nm laser excitation (20 mW laser power in the sample plane, 2 s accumulation time) using a 40× objective lens MPlanFL (Nikon, Tokyo, Japan) with a numerical aperture of 0.75. According to the specifications provided by the manufacturers, the transverse and longitudinal optical resolutions for the used equipment and parameters were <1 µm and <10 µm, respectively. The spectral resolution was in the range of 1 to 1.5 cm^−1^ for both spectrometers. In total, 10 to 20 spectra were obtained at different points of each sample at room temperature under equal conditions for two excitation wavelengths to form two datasets (532 nm and 785 nm) with equal numbers of spectra as well as a combined dataset (532 + 785) with equal number of paired 532 nm and 785 nm spectra. All datasets were divided into three classes: BCC, normal skin (Normal), and SCC.

**Figure 8:**
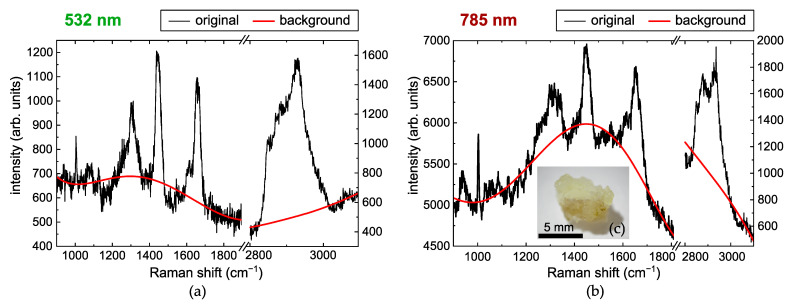
Examples of original acquired spectra (black curves) and estimated fluorescence background signals (red curves) of the basal cell carcinoma sample for (**a**) 532 nm and (**b**) 785 nm excitation wavelengths, and (**c**) a photo of the sample.

The acquired spectra were preprocessed using MATLAB (R2022b, MathWorks, Natick, MA, USA) implementations of the Vancouver Raman Algorithm [73,74,75,76,77] (modified multi-polynomial baseline fitting) to remove tissue fluorescence background, and the Savitzky–Golay filter was used to reduce noise. The wavenumber ranges from 900 to 1800 cm^−1^ and from 2800 to 3100 cm^−1^ were considered for further analysis. Examples of the raw spectra and the estimated background signals for both excitation wavelengths are given in Figure 8.

To evaluate the appropriateness of various spectral features for differentiation between the normal skin and the tumors, we used several methods of feature extraction. First, we performed the PCA of the processed spectra independently for the two datasets acquired at 532 nm and 785 nm excitation wavelengths. Prior to the PCA, the spectra were normalized, i.e., divided by the maximum Raman intensity in the 1440–1450 cm^−1^ band to reduce the influence of tissue heterogeneity, respectively, and allowed us to reveal relative changes in the content of different lipid components and obtain proteins/lipids ratios [6,18,78]. The calculated PCA coefficients were used to estimate the classification performance by means of quadratic discriminant analysis in MATLAB Classification Learner with default settings. To find the best set of *N* features (N=2…5), we tried all possible combinations of *N* PCA coefficients and chose the sets with highest TPR values, e.g., the best set of three features (N=3) extracted from the 532 nm dataset included second, fourth, and seventh components (enumerated according to the eigenvalues in descending order) labeled PCA_2, PCA_4, and PCA_7, respectively. For the merged dataset, we considered all combinations of PCA features from both 532 nm and 785 nm datasets, e.g., the highest TPR for the merged dataset was achieved using PCA_2 and PCA_7 from 532 nm and PCA_8 from 785 nm. This method is hereafter referred to as ‘PCA spectra’.

Second, we replaced each spectra with an array *I* of its peak intensities in characteristic Raman bands representing the main tissue components listed in the next section. We noted each band according to the mean position of its peak; thus, an element of the array *I* corresponding to the 1265–1271 cm^−1^ band is noted as $I(1268\;{\mathrm{cm}}^{-1})$. In some cases, the mean position is different for the two excitation wavelengths, which leads to different labels of the same band, e.g., $I(1445\;{\mathrm{cm}}^{-1})$ and $I(1442\;{\mathrm{cm}}^{-1})$ for 532 and 785 nm, respectively. When referring to such bands for both wavelengths in the next sections, we use the notation for 532 nm to avoid unnecessary duplication. To normalize each array, we divided its elements by $I(1445\;{\mathrm{cm}}^{-1})$ and obtained an array *R* of ratios: $R\left(1268\;{\mathrm{cm}}^{-1}\right)=I\left(1268\;{\mathrm{cm}}^{-1}\right)/I\left(1445\;{\mathrm{cm}}^{-1}\right)$. We similarly applied the PCA to the datasets consisting of the arrays *R* and chose the sets of *N* features with the highest overall TPR values (further referred to as ‘PCA bands’). Next, we used the ratios *R* as spectral features and tried all possible combinations of *N* ratios, e.g., the best set of three ratios from the 532 nm dataset contained $R(1268\;{\mathrm{cm}}^{-1})$, $R(1336\;{\mathrm{cm}}^{-1})$, and $R(2850\;{\mathrm{cm}}^{-1})$. These results are labeled as ‘Selected bands’. Finally, we repeated the aforementioned analysis using different bands for normalization to consider all possible ratios of the intensities in the main Raman bands.

## 4. Conclusions

In this study, we acquired in vitro Raman spectra of human skin tissue samples, including healthy skin, BCC, and SCC, at 532 and 785 nm laser excitation wavelengths and processed them to find the most representative spectral features for differentiation between the three classes of the samples. The PCA and the correlation analysis demonstrated that ≈25 main Raman bands can be divided into two groups to characterize the lipid–protein proportion, while the protein-associated bands (including bands of nucleic acids) can be further subdivided into two groups depending on their intensification in malignancies; this division constituted a basis for the differentiation. Using intensity ratios of several properly selected Raman bands provided a similar classification rate as an equal number of the most suitable PCA features, yielding up to 80%, 88%, and 95% overall TPR (accuracy) for two, three, and five features, respectively. The intensity ratios of the bands at 1268, 1336, and 1445 cm^−1^ appeared to be the most reliable criteria for the three-class differentiation at 532 nm excitation, whereas the bands from the higher wavenumber region (2850, 2880, and 2930 cm^−1^) were a robust measure of the increased protein/lipid ratio in the tumors at both excitation wavelengths. The excessive intensity of some protein-related bands in the spectra of the SCC samples at 532 nm, which allows distinguishing between them and the other two classes but does not take place at 785 nm excitation, can hardly be explained by massive keratinization and may be caused by resonant-enhanced Raman scattering of the heme proteins, some of which were proved to be markers of cancer in other studies. Thus, the acquisition of Raman spectra of skin tissues using 532 nm excitation can reveal new biochemical indicators of tumor development and improve their detection at early stages, complementing commonly used 750–850 nm lasers in the development of multi-wavelength excitation Raman spectroscopic techniques. In particular, selecting three bands from the merged (532 + 785) dataset made it possible to increase the accuracy to 87% for the three classes and reach specificities for BCC + SCC equal to 87% and 81% for the sensitivities of 95% and 99%, respectively. Further investigations can be related to the evaluation of these methods using a larger set of samples, including melanoma, pigmented nevus, keratoma, and actinic keratosis, and a comparison between in vivo and in vitro measurements. We believe that multi-wavelength excitation Raman microspectroscopy can be a versatile non-invasive tool for both research of the processes in malignant skin tumors, as well as other forms of cancer, and their early diagnosis.

## Figures and Tables

**Table 1:** Peak positions of main Raman bands in the Raman active components at 532 and 785 nm.

Raman Peaks, ${\mathrm{cm}}^{-1}$		Band Assignments	Components	References
532 nm *	785 nm *			
936–940	936–940	ν(C–C) **, AA *** side chain vibrations	proteins (α-helix), collagen (Pro, Val)	[37,38,39]
1000–1005	1000–1005	ν(C–C) aromatic ring breathing, Phe; $\rho ({\mathrm{CH}}_{3})$, carotenoids	proteins (Phe), collagen, elastin, keratin, carotenoids	[3,16,18,37]
1031–1035	1031–1037	δ(C–H) in-plane bending, $\delta ({\mathrm{CH}}_{2}{\mathrm{CH}}_{3})$, Phe	proteins (Phe, Pro)	[18,40]
1060–1063	1060–1064	ν(C–C) skeletal (trans)	lipids, ceramide	[20,40,41]
1080–1084	1079–1085	ν(C–C) or ν(C–O) skeletal (gauche), phospholipids; $\nu ({\mathrm{PO}}_{2})$, nucleic acids	phospholipids, triolein, nucleic acids	[16,20,40]
1126–1130	1126–1130	ν(C–C) skeletal (trans), phospholipids; ν(C–N), proteins; $\nu_{22}$, heme	phospholipids, ceramide, Cyt b, Cyt c, Mb	[3,16,20,28,39,40,42]
1155–1160	–	ν(C–C) and ν(C–N), proteins and carotenoids	proteins, carotenoids, glycogen	[16,40,43]
–	1175–1180	β(C–H) in-plane, Tyr	proteins (Tyr, Ct), G	[40,44]
1205–1210	1208–1210	ν(C–C6$H_{5}$),Phe,Trp	proteins (Phe, Trp)	[16,40,45]
1248–1250	1248–1251	Amide III (β-sheet, unordered), proteins	proteins, collagen, elastin, A, Ct, T	[20,39,40]
1265–1271	1265–1271	Amide III (α-helix), ν(C–N), β(N–H) in-plane, proteins; δ(=C–H), lipids	proteins (α-helix), collagen, elastin, lipids (unsaturated)	[20,38,39,40]
1300–1302	1300–1302	$\tau ({\mathrm{CH}}_{2},{\mathrm{CH}}_{3})$	lipids, triolein, proteins (aliphatic AA), A, Ct, Cyt b	[16,20,28,40,42,46]
1314–1317	1314–1317	ν(C–N), ν(C–H), proteins; $\nu_{21}$, heme	proteins (Trp), collagen, G, Cyt c	[28,39,47]
1336–1340	1336–1340	ν(C–N), β(N–H), ν(C–H), proteins; $\omega ({\mathrm{CH}}_{2},{\mathrm{CH}}_{3})$, nucleic acids; $\nu_{41}$, heme	proteins (aliphatic AA, Trp), elastin, A, G, Cyt b, Mb	[16,20,28,40,48]
1393–1396	1393–1396	$\omega ({\mathrm{CH}}_{2})$, ρ(C–H), leucine; $\nu_{20}$, heme	leucine, Cyt c, Mb	[16,28,40,49]
1440–1454	1440–1450	δ(C–H), proteins; $\alpha ({\mathrm{CH}}_{2},{\mathrm{CH}}_{3})$, lipids	proteins (aliphatic AA), keratin, collagen, elastin, lipids, triolein, ceramide	[16,20,39,40,41]
1513–1516	–	ν(C=C)	carotenoids	[8,16,43]
1554–1559	1552–1556	Amide II, ν(C=C), Trp; ν(C=C), $\nu_{37}$, heme	proteins (Trp), Deoxy-Mb	[3,16,40,45]
1584–1589	1582–1589	β(C=C), ν(C=C) olefinic, proteins (Phe); $\nu_{37}$, heme	proteins (Phe), Cyt c, Oxy-Mb	[3,11,16,27,40,50,51]
1604–1606	1604–1606	δ(C=C) in-plane, proteins (Phe, Tyr); $\delta ({\mathrm{NH}}_{2})$, Ct; $\nu_{19}$, heme	proteins (Phe, Tyr), Ct, Deoxy-Mb	[16,40,44]
1651–1659	1652–1656	Amide I (α-helix), ν(C=O), proteins; ν(C=C), lipids	proteins, collagen, elastin, keratin, lipids (unsaturated), triolein	[3,13,16,20,38,39,40,41,45]
1743–1745	1743–1745	ν(C=O)	lipids (esters), phospholipids	[3,16,52]
2849–2855	2850–2854	ν(=CH_2_) symmetric, ν(C–H)	lipids	[41,42,53,54]
2879–2885	2880–2884	ν(=CH_2_) asymmetric, ν(C–H)	lipids, proteins	[11,41,55,56]
2929–2934	2925–2933	ν(=CH_3_) symmetric, δ(C–H)	proteins (aromatic AA), nucleic acids, lipids	[41,53,54,56,57]
3008–3012	3010–3013	ν(=C–H) olefinic	lipids (unsaturated)	[11,41,56,57]
3040–3042	3040–3042	ν(C–H)	proteins (Phe)	[57]
3060–3063	3060–3064	Amide B, ν(N–H), ν(C–H)	proteins (aromatic AA)	[11,42,57,58]

* Excitation wavelength. ** α, scissoring; β, bending; δ, deformation; ν, stretching; ρ, rocking; τ, twisting; ω, wagging. *** Aromatic amino acids (AA): Phe, phenylalanine; Trp, tryptophan; Tyr, tyrosine. Aliphatic AA: Pro, proline; Val, valine. Nucleic acids: A, adenine; Ct, cytosine; G, guanine, T, thymine. Heme proteins: Hb, hemoglobin; Mb, myoglobin; Cyt, cytochromes (b and c).

**Table 2:** Classification rates for various numbers of spectral features and different calculation methods.

*N* *	Data **	True Positive Rate (TPR), %											
		PCA Spectra ***				PCA Bands ***				Selected Bands ***			
		All	BCC	Normal	SCC	All	BCC	Normal	SCC	All	BCC	Normal	SCC
2	532	79.5	72.2	76.3	92.5	79.1	59.5	87.1	91.0	77.4	68.4	81.7	82.1
2	785	70.3	48.1	75.3	89.6	67.0	34.2	87.1	77.6	72.0	48.1	81.7	86.6
2	532 + 785	79.5	72.2	76.3	92.5	79.1	59.5	87.1	91.0	77.4	68.4	81.7	82.1
3	532	87.9	87.3	83.4	94.0	86.2	74.7	90.3	94.0	82.9	72.2	88.2	88.1
3	785	76.2	54.4	87.1	86.6	75.3	59.5	81.7	85.1	75.7	54.4	83.9	89.6
3	532 + 785	87.9	82.3	88.2	94.0	86.2	76.0	89.3	94.0	86.6	78.5	90.3	91.0
4	532	90.4	87.3	88.2	97.0	88.7	78.5	93.6	94.0	87.4	79.8	89.3	94.0
4	785	81.6	73.4	85.0	86.6	78.7	55.7	89.3	91.0	80.3	68.4	85.0	88.1
4	532 + 785	91.2	91.1	89.3	94.0	90.8	86.1	91.4	95.5	89.5	82.3	94.6	91.0
5	532	95.0	93.7	95.7	95.5	92.1	84.8	94.6	97.0	91.6	83.5	93.5	98.5
5	785	85.8	83.5	87.1	86.6	81.6	64.6	88.2	92.5	83.3	68.4	87.1	95.5
5	532 + 785	95.0	93.7	95.7	95.5	93.3	91.1	93.6	95.5	92.5	88.6	93.5	95.5

* *N*, number of spectral features. ** Dataset acquired at the specified excitation wavelength (532 nm, 785 nm) or merged dataset (532 + 785). *** Method for calculating spectral features: PCA spectra, principle component analysis (PCA) using normalized Raman spectra; PCA bands, PCA using normalized peak intensities (ratio of intensities) for main Raman bands; Selected bands, normalized peak intensities (ratio of intensities) for selected Raman bands.

**Table 3:** Selected sets of Raman bands with the highest classification rates.

*N* *	Raman Bands, ${\mathrm{cm}}^{-1}$		True Positive Rate, %			
	532 nm	785 nm	All	BCC	Normal	SCC
2	1268, 1336	× **	77.4	68.4	81.7	82.1
2	1248, 1336	×	76.6	63.3	77.4	91.0
2	×	2880, 3062	72.0	48.1	81.7	86.6
2	×	2880, 3040	71.6	46.8	82.8	86.6
3	1268, 1336, 2850	×	82.9	72.2	88.2	88.1
3	1062, 1248, 1336	×	82.4	64.6	86.0	98.5
3	1248, 1268, 1336	×	82.4	73.4	86.0	88.1
3	1248, 2850, 2930	×	82.4	69.6	83.9	95.5
3	×	2850, 2880, 3040	75.7	54.4	83.9	89.6
3	×	940, 2850, 2930	75.3	54.4	81.7	91.0
3	×	2850, 2880, 3062	74.9	53.2	82.8	89.6
3	1268, 1336	2880	86.6	78.5	90.3	91.0
3	1268, 1336	3062	84.5	76.0	91.4	85.1
4	1268, 1336, 1585, 2850	×	87.5	79.8	89.3	94.0
4	1248, 1268, 1336, 3062	×	86.6	79.8	87.1	94.0
4	1268, 1336, 1604, 3040	×	86.6	77.2	89.3	94.0
4	1268, 1336, 1604, 3062	×	86.6	72.2	94.6	92.5
4	×	940, 1393, 2880, 3062	80.3	68.4	85.0	88.1
4	×	940, 1654, 2850, 2930	79.1	64.6	81.7	92.5
4	×	940, 2850, 2880, 3040	79.1	60.8	83.9	94.0
4	1248, 1268, 1336	1336	89.5	82.3	94.6	91.0
4	1062, 1268, 1336	3062	88.7	82.3	94.6	88.1
5	1248, 1585, 1604, 2850, 2930	×	91.6	83.5	93.5	98.5
5	1268, 1336, 1604, 2880, 3062	×	91.2	83.5	94.6	95.5
5	×	1208, 1393, 1654, 2850, 3062	83.3	68.4	87.1	95.5
5	×	1208, 1393, 1654, 2850, 3040	82.9	69.6	87.1	92.5
5	×	940, 1336, 1393, 1654, 2850	82.1	69.6	86.0	91.0
5	×	940, 1393, 1654, 2880, 3062	82.1	68.1	87.1	91.0
5	1004, 1268, 1336, 1604	940	92.5	88.6	93.5	95.5
5	1248, 1268	940, 2850, 2930	92.5	84.8	94.6	98.5

* *N*, number of spectral features; ** × indicates that the data acquired at this excitation wavelength were not used.

**Table 4:** Sets of three selected Raman bands with the highest classification rates for data normalization using different bands.

Norm. Band, * ${\mathrm{cm}}^{-1}$		Selected Bands, ${\mathrm{cm}}^{-1}$		True Positive Rate, %			
532 nm	785 nm	532 nm	785 nm	All	BCC	Normal	SCC
1336	× **	1208, 1268, 1445	×	84.9	78.5	84.9	92.5
1268	×	1208, 1336, 1445	×	84.1	70.8	87.1	95.5
2880	×	1248, 1268, 1336	×	83.7	72.2	91.4	86.6
2930	×	1268, 1336, 2880	×	82.9	69.6	84.9	95.5
1445	×	1268, 1336, 2850	×	82.9	72.2	88.2	88.1
×	2930	×	940, 1654, 2880	79.9	72.2	76.3	94.0
×	2850	×	940, 1393, 1654	78.2	58.2	88.2	88.1
×	2880	×	940, 1745, 2930	77.8	60.8	81.7	92.5
×	1082	×	940, 2850, 2930	77.0	68.4	76.3	88.1
×	1442	×	2850, 2880, 3040	75.7	54.4	83.9	89.6
1268	2880	1248	940, 2930	87.5	78.5	89.3	95.5
1336	2850	1268, 2850	3040	87.5	77.2	91.4	94.0
1336	2880	1248, 1268	3040	87.0	78.5	89.2	94.0
2930	2930	2880	940, 2850	86.6	82.3	82.8	97.0
1445	1442	1268, 1336	2880	86.6	78.5	90.3	91.0

* The band used for normalization. ** × indicates that the data acquired at this excitation wavelength were not used.

## Data Availability

The data supporting the reported results can be obtained from the authors.

## Supplementary Materials

The following supporting information can be downloaded at: https://www.mdpi.com/article/10.3390/ijms25137422/s1.
