# Supplementary material for: Multi-Wavelength Raman Differentiation of Malignant Skin Neoplasms
Source: Int J Mol Sci. 2024 Jul 6;25(13):7422. doi: 10.3390/ijms25137422 (PMC11242141; doi:10.3390/ijms25137422)
Supplement: Supplementary file 1 [file ijms-25-07422-s001.zip › ijms-3048277-supplementary.pdf]

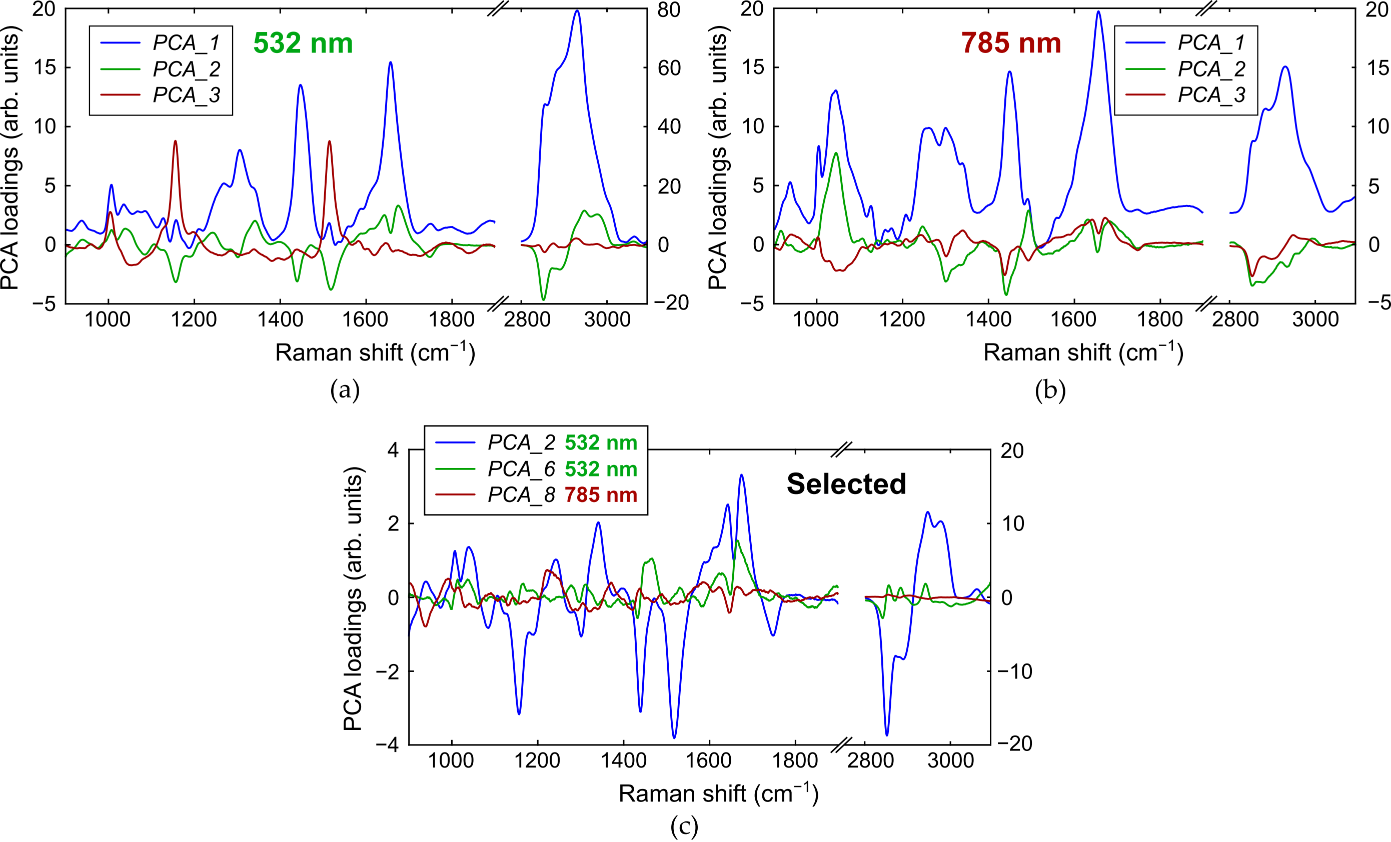

Figure S1. Principal components analysis (PCA) features (loadings) calculated from the datasets acquired with (**a**) 532 nm and (**b**) 785 nm excitation. (**c**) The set of three PCA features taken from both datasets that provides the best overall true positive rate (TPR).
